# Transcriptional Profiling of Botulinum Neurotoxin Type A-Related Molecular Components in Primary Human Schwann Cells

**DOI:** 10.3390/toxins18080321

**Published:** 2026-07-24

**Authors:** Oscar Sánchez-Carranza, Claudia Jatzke, Andreas Gravius, Jens Nagel

**Affiliations:** Merz Therapeutics GmbH, Eckenheimer Landstraße 100, 60318 Frankfurt am Main, Germany; claudia.jatzke@merz.de (C.J.); andreas.gravius@merz.de (A.G.)

**Keywords:** botulinum neurotoxin, BoNT/A, Schwann cells, human, paclitaxel, pain

## Abstract

Schwann cells (SC) myelinate peripheral axons and orchestrate nerve regeneration after injury by switching between myelinating, proliferative and repair states. Evidence suggests that Botulinum Neurotoxin Type A (BoNT/A) influences SC biology, potentially supporting nerve repair and pain relief in peripheral neuropathic pain (PNP) models. However, BoNT/A receptor and target expression in human SC (hSC) remains poorly explored. Here, this translational gap was addressed by transcriptionally profiling genes encoding BoNT/A-relevant receptors and targets in primary hSC and testing whether paclitaxel evokes hSC phenotype plasticity in vitro based on changes in gene expression. Primary hSC were isolated, cultured, and treated with paclitaxel or vehicle, followed by RT-qPCR profiling of hSC markers and BoNT/A receptor/targets genes. Untreated hSC expressed moderate *NGFR* and *S100β*, with low *MBP* levels, suggesting a non-myelinating state profile. Transcripts encoding the BoNT/A receptor machinery (SV2A, SYT1) and the target SNAP25 were detectable at moderate levels. Paclitaxel induced changes in gene expression: *SV2A* and *SYT1* decreased (up to two-fold), whereas *SNAP25* and *MBP* increased, accompanied by reduced *NGFR*, indicating a shift toward a more differentiated transcriptional state. These data indicate hSC transcriptional plasticity in vitro and provide transcriptional evidence for the expression of BoNT/A-related molecular components in non-neuronal human cells.

## 1. Introduction

Peripheral neuropathic pain (PNP) is a debilitating condition estimated to affect 10% of the population [1,2] and is caused by a lesion or disease of the peripheral somatosensory system [3] decreasing patient’s quality of life. PNP results from nerve damage caused by infections (e.g., postherpetic neuralgia), genetic or metabolic diseases such as diabetic neuropathy, chemical injury by chemotherapy agents, as well as traumatic or post-operative nerve injury [4]. Nerve damage leads to significant changes affecting sensory neurons and non-neuronal cells, such as Schwann cells (SC). Schwann cells are the glia cells of the peripheral sensory system, providing nerve support and assuring fast and reliable conductance in Aβ- and Aδ-fibers. They also contribute to ensheathment of C-fibers into Remak bundles. Importantly, SC participate during nerve repair and regeneration following injury, enabling transitions from a myelinating state to a repair phenotype and back to mature SC along regenerated axons [5,6].

Paclitaxel treatment is frequently associated with the development of chemotherapy-induced peripheral neuropathy (CIPN) [7]. It induces nerve injury simultaneously across different peripheral nervous system (PNS) structures and pathways, affecting both neuronal and non-neuronal cells [7,8]. These changes ultimately result in axonal and myelin loss in the peripheral nerves and lead consequently to the development of sensory symptoms in patients including allodynia and hyperalgesia, among other symptoms [9,10,11]. To date, research on paclitaxel-induced peripheral neuropathy has largely focused on its effects on sensory neurons, particularly on Aβ-, Aδ- and, in minor degree, C-fibers. In contrast, relatively few studies have addressed its impact on SC. This gap is even more pronounced in human systems, where the response of human SC (hSC) to paclitaxel remains poorly characterized [12,13,14,15].

Botulinum neurotoxin type A (BoNT/A) is a clostridial neurotoxin that has emerged as a promising potential treatment option to address peripheral neuropathic pain (PNP) [4,16,17,18,19]. Its canonical neuronal mechanism of action (MoA) involves BoNT/A binding to polysialoganglioside (PSG), synaptotagmin 1 (SYT1) and synaptic vesicle glycoprotein 2 (SV2), which mediate neurotoxin recruitment and internalization [20,21]. Once in the cytoplasm, BoNT/A cleaves the Synaptosomal-Associated Protein, 25kDa (SNAP-25) resulting in the inhibition of neurotransmitter release. However, the mechanisms underlying its analgesic effects in PNP are not yet fully understood [22,23]. Using rodent models, it has been proposed that the analgesic effects of BoNT/A in PNP may arise partially by promoting early functional recovery. BoNT/A may contribute by recruiting SC, enhancing their proliferation, and promoting repair process at the injury site [24,25,26]. Moreover, cleaved SNAP25 has been observed in SC following intraplantar BoNT/A injection in mice [27]. Marinelli et al. reported that BoNT/A reduced acetylcholine release in isolated and cultured mouse SC in vitro and triggered SC proliferation, which may help explain its regenerative effects observed in rodent models [27]. These findings support a multimodal MoA of BoNT/A and suggest that SC may express BoNT/A-related molecular components. However, it remains unclear whether genes encoding components of the canonical BoNT/A pathway are expressed in hSC.

Here, it was investigated whether primary hSC express transcripts encoding BoNT/A-relevant molecular components [20] and whether paclitaxel treatment induces hSC phenotypic plasticity in vitro, as reflected by transcriptional changes of SC marker genes. Our findings provide a translational and transcriptional basis for future studies investigating how BoNT/A may act in non-neuronal human cells and contribute to its therapeutic effects in PNP.

## 2. Results

### 2.1. Paclitaxel Compromises hSC Viability

**Figure 1:**
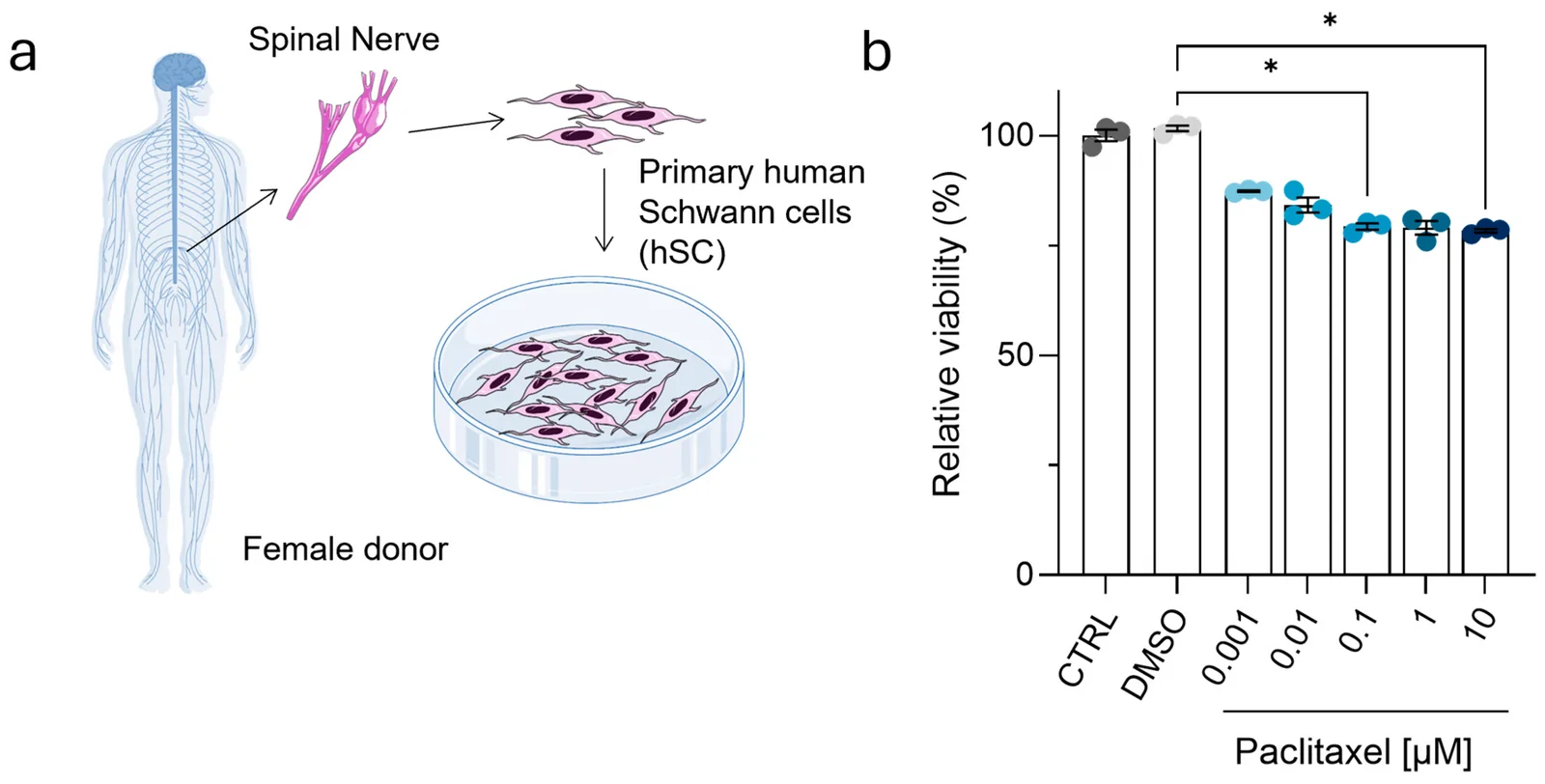
Paclitaxel reduces hSC viability in vitro. (**a**) Cartoon representing the origin of primary hSC used in this study. (**b**) Bars plot showing the percentages of relative viability of primary hSC subjected to increasing concentrations of paclitaxel. Viability was calculated relative to control untreated cells (CTRL). Kruskal–Wallis, Dunn’s test; * *p* < 0.05. Each dot represents a technical replicate (*n* = 3). Data are presented as the mean ± standard error of the mean. Cell viability was determined at cell passage 5.

First, it was tested whether paclitaxel affected hSC viability in vitro. Paclitaxel was selected because, unlike platinum derivates (e.g., cisplatin or oxaliplatin), it has been shown to induce SC dedifferentiation in mice without causing mitochondrial dysfunction [14]. Primary hSC were incubated with paclitaxel at different concentrations, and the cell viability was quantified 48 h post-treatment. Figure 1 shows that the hSC viability was reduced by 15–20% across the selected paclitaxel concentration range. This reduction did not reach statistical significance at 1 nM, 10 nM and 1 μM paclitaxel. In contrast, treatment with 100 nM and 10 μM paclitaxel resulted in a significant reduction in cell viability compared with DMSO-treated cells. No differences in viability were observed between DMSO-treated cells and untreated cells, indicating that the vehicle alone did not affect the hSC viability. Thus, paclitaxel can induce cytotoxic effects in primary hSC in vitro.

### 2.2. Paclitaxel Induces Transcriptional Changes in hSC Phenotype-Associated Markers

Previously, paclitaxel was reported to induce phenotypic changes in mouse SC, shifting them from a differentiated cell phenotype characterized by low expression of the p75 neurotrophin receptor (p75NTR, encoded by the *Ngfr* gene) and high expression of myelin basic protein (MBP) to a dedifferentiated phenotype characterized by high p75NTR and low MBP expression [14]. Therefore, the phenotypic profile of primary hSC under the present in vitro conditions was first determined by examining the expression of characteristic maker genes. Based on threshold cycle (C_t_) values (Table 1), untreated primary hSC expressed moderated levels of transcript encoding S100 Calcium Binding Protein β (*S100β*), a canonical SC marker. In addition, cells showed moderate expression of *NGFR* and low expression *MBP*, consistent with a non-myelinating-associated transcriptional phenotype.

**Figure 2:**
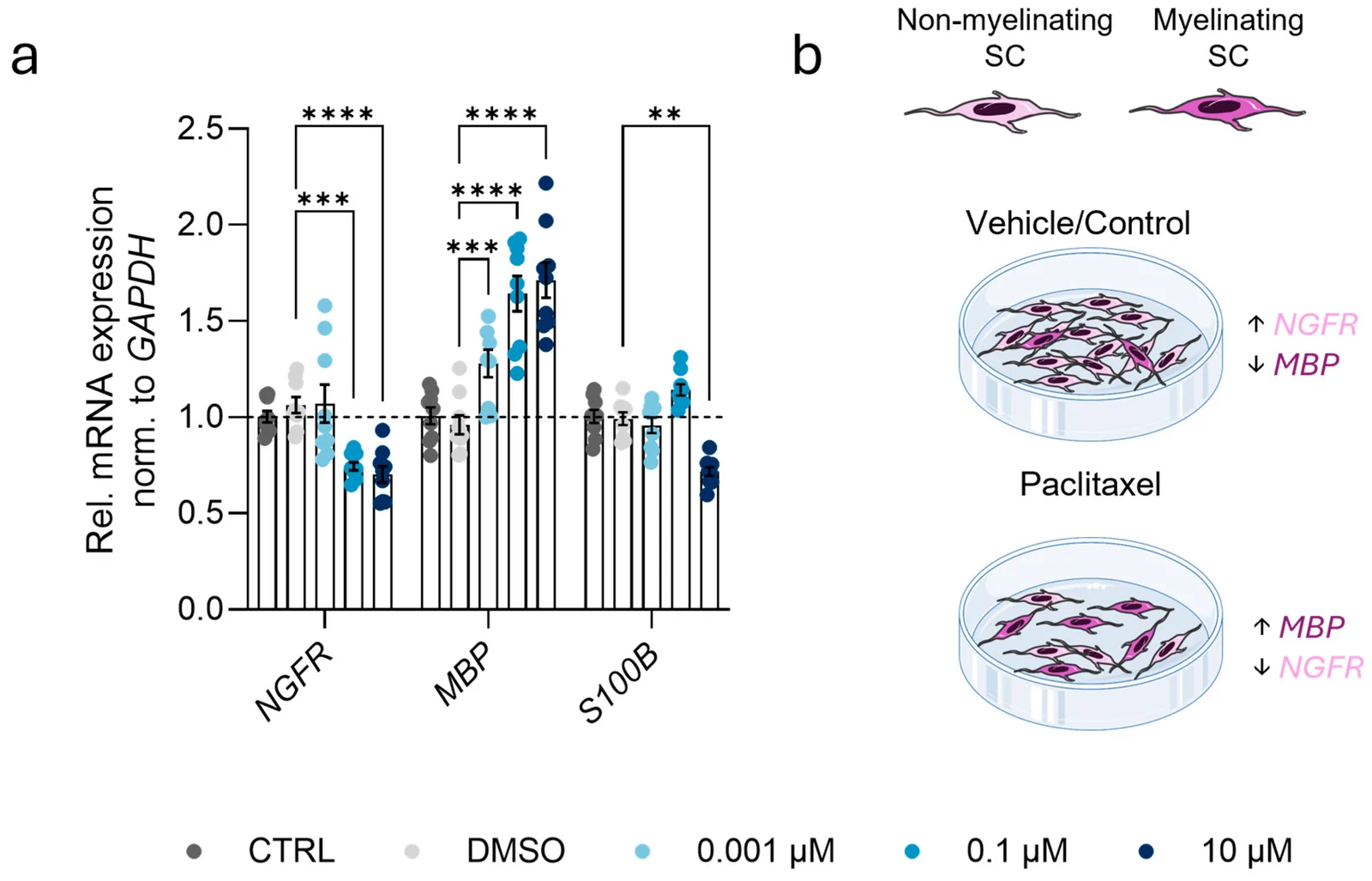
Paclitaxel evokes transcriptional changes in hSC phenotype-associated markers. (**a**) Relative mRNA expression levels of SC markers in primary hSC upon 48 h of paclitaxel treatment. The analyzed genes were normalized to *GAPDH* expression levels for each sample, and 2^−ΔΔCt^ values were calculated using the untreated CTRL group as reference. Each dot represents a technical replicate. Data obtained from three independent experiments (*n* = 3), each performed in technical triplicate. Mean ± standard error of the mean. Two-way ANOVA; Dunnet test, ** *p* = 0.002; *** *p* < 0.001; **** *p* < 0.0001. (**b**) Schematic representation indicating the transcriptional phenotypic shift in primary hSC post-paclitaxel treatment.

Upon 48 h of paclitaxel treatment, primary hSC displayed dose-dependent transcriptional changes. As shown in Figure 2, treatment with 0.1 and 10 μM paclitaxel significantly reduced the relative *NGFR* transcript levels by approximately 30% compared with DMSO-treated cells. In contrast, paclitaxel increased the relative *MBP* mRNA expression by up to 1.7-fold. No significant changes were observed in the relative *S100β* expression, except at 10 µM paclitaxel, where a significant reduction of ~30% was observed. These changes are supported by the C_t_ values (Supplementary Table S1). To note, the C_t_ values for *GAPDH* were largely comparable between untreated and treated cells. However, a small increase was observed at 10 μM paclitaxel relative to DMSO-treated cells (Supplementary Table S1). Thus, under the present experimental conditions, paclitaxel induces transcriptional changes in primary hSC, including reduced *NGFR* and increased *MBP* expression, consistent with a shift toward a more differentiated transcriptional state.

### 2.3. Primary hSC Express Transcripts Encoding Relevant Molecules Associated with the Canonical BoNT/A MoA

**Figure 3:**
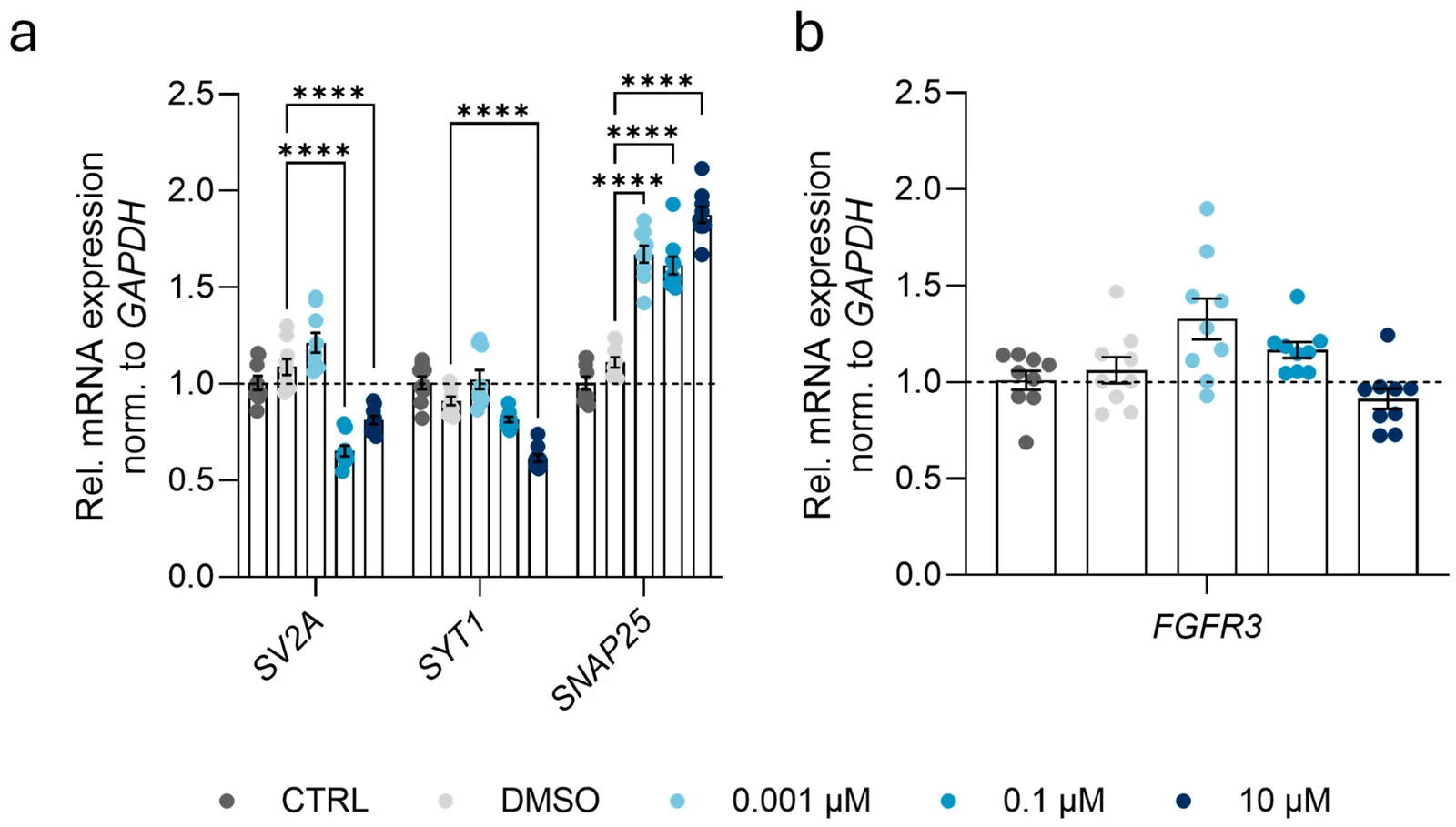
Paclitaxel modulates transcripts encoding molecules associated with the canonical BoNT/A MoA in primary hSC. (**a**) Histogram showing relative mRNA expression levels encoding relevant canonical BoNT/A molecular components in primary hSC upon 48 h of paclitaxel treatment. The analyzed genes were normalized to *GAPDH* expression levels for each sample, and 2^−ΔΔCt^ values were calculated using the untreated CTRL group as reference. Two-way ANOVA; Dunnet test, **** *p* < 0.0001. (**b**) Bar plot showing that expression of *FGFR3* transcripts were not altered in primary hSC post-paclitaxel treatment. Kruskal–Wallis; Dunn’s test. Each dot represents a technical replicate. Data obtained from three independent experiments (*n* = 3), each performed in technical triplicate. Mean ± standard error of the mean.

The expression of mRNAs encoding relevant canonical BoNT/A-related molecular components was subsequently assessed in primary hSC. Based on the C_t_ values, untreated primary hSC expressed moderate transcript levels of genes encoding BoNT/A-related receptors SV2A and SYT1, as well as of the BoNT/A target SNAP25 (Table 1). The expression of *SV2A*, *SYT1* and *SNAP25* was then examined upon paclitaxel treatment. As shown in Figure 3a, paclitaxel at 0.1 and 10 μM reduced *SV2A* expression up to two-fold after a 48 h treatment. Similar effects were observed for *SYT1* expression at 10 μM compared with DMSO-treated cells. In contrast, *SNAP25* expression was significantly upregulated at all concentrations tested (Figure 3a). The C_t_ values (Supplementary Table S1) support the direction of the normalized expression changes and provide additional information on the transcript abundance before normalization. Additionally, the expression of the proposed non-canonical BoNT/A receptor Fibroblast Growth Factor Receptor 3 (FGFR3) was assessed [28]. *FGFR3* transcripts were detected at low abundance based on the C_t_ values (Table 1), and their expression was not significantly altered by paclitaxel (Figure 3b). Thus, these findings show that primary hSC express transcripts encoding molecules associated with the canonical BoNT/A MoA and that their expression is modulated by paclitaxel treatment.

## 3. Discussion

Exposure to clinically relevant concentrations of paclitaxel [29,30]—used here as an in vitro model of CIPN—altered the transcriptional profile of primary hSC, indicating that these cells retain phenotypic plasticity under the present experimental conditions. Moreover, it was shown that primary hSC express transcripts encoding molecules associated with the canonical BoNT/A MoA. These findings provide a basis for future studies investigating the BoNT/A MoA in a human non-neuronal context, helping to bridge a translational gap in the understanding of BoNT/A in PNP and its potential role in nerve regeneration and analgesia following nerve injury. Our data demonstrate that untreated primary hSC expressed moderate detectable transcripts encoding the BoNT/A receptors SV2A and SYT1, as well as cytoplasmatic target SNAP25. Paclitaxel treatment induced transcriptional changes in both SC phenotypic markers and BoNT/A-related component genes. These findings extend previous observations from rodent studies [24,25,27,31,32] and provide transcriptional evidence that human non-neuronal cells, such as hSC, express genes encoding BoNT/A-relevant molecular components, supporting further investigation of potential BoNT/A-related mechanisms in this cell type.

The use of paclitaxel, as an in vitro CIPN model, has been widely studied in different cell types, including sensory neurons and SC in rodents. The selected paclitaxel range was based on clinical pharmacokinetic data, previous in vitro studies, and an initial titration experiment in primary hSC aimed at identifying the concentrations suitable for assessing both cell viability and transcriptional responses [14,29,30,33]. Previous studies demonstrated that both sensory neurons and SC react to paclitaxel treatment [14,34]. Interestingly, Imai et al. showed that myelinating SC increased p75NTR and reduced MBP markers at the transcript and protein level after taxane exposure in vitro [14]. Even though our data indicate that primary hSC respond to paclitaxel and undergo transcriptional changes as part of a pathological stress response, the direction of this shift differs from that reported by Imai et al. One possible explanation is that, in an initially immature/non-myelinating phenotype, paclitaxel treatment changes the direction of the response. Alternatively, not mutually exclusive, it is possible that paclitaxel affects preferentially the *NGFR*-enriched immature subpopulation, resulting in a relative enrichment of cells with a more differentiated transcriptional level, including increased *MBP* expression. This interpretation is supported by the observation that paclitaxel significantly reduced cell viability (Figure 1). However, because 10 μM reduced hSC viability and altered transcript C_t_ values in all genes analyzed (Supplementary Table S1), the results obtained at this concentration should be interpreted cautiously and should not be considered a direct representation of typical clinical exposure. Even though the contribution of SC to nerve repair after injury is well established, it remains unclear which SC subtype is implicated in this dynamic process and whether their contribution differs between proximal and distal regions relative to the injury site, particularly under disease conditions [15,35,36]. Therefore, further analysis quantifying the proportion of myelinating and non-myelinating hSC phenotypes in vitro would be needed to support these hypotheses. Furthermore, because our study was limited to a small marker panel at the mRNA level, this remains speculative, and further studies combining single cell transcriptomics with proteomics would better define these phenotypic changes in healthy and disease environments [37,38,39].

Understanding the mechanisms that regulate nerve regeneration and repair may help identify new therapeutic strategies for patients after nerve injury. Marinelli et al. were the first to demonstrate that, following nerve injury, BoNT/A treatment enhanced SC proliferation and migration to the injury site, where an accelerated functional recovery was parallelly observed in rodent models [24,27]. These findings were later supported by independent groups [25,31,32]. Additionally, Marinelli et al. showed direct BoNT/A effects on isolated mouse SC in vitro, including increased cell proliferation and inhibition of acetylcholine release [27]. In the same study, the authors reported cleaved SNAP25 immunoreactivity that overlapped with SC markers [27], suggesting that SC express the molecular machinery to uptake BoNT/A, as further discussed in [40]. However, whether SC internalize BoNT/A by the canonical or non-canonical pathways remained unclear. Moreover, most of the available evidence comes from rodent models, and the molecular basis of BoNT/A action in hSC remains largely unexplored.

In neurons, BoNT/A canonical uptake depends on interactions with PSG, SYT1 and SV2 for neurotoxin recruitment and internalization culminating in SNAP25 cleavage [20,23,41]. Additionally, FGFR3 has been proposed as a non-canonical BoNT/A (co-) receptor, although it was not detected in human dorsal root ganglia (DRG) neurons [28,42]. To our knowledge, this is the first report demonstrating that primary hSC express transcripts encoding molecular machinery relevant to the canonical BoNT/A MoA in vitro, supporting the translational relevance of this model for future mechanistic studies. Under the present experimental conditions, *FGFR3* transcripts were detected at low abundance, suggesting the hSC may uptake BoNT/A through the canonical pathway. This interpretation is consistent with a recent finding showing that FGFR3 expression was undetectable in human DRG and tibial nerves, whereas its expression was observed in human spinal cord tissue [42]. Even though Chamessian et al. did not carry out co-staining or the direct identification of expression of FGFR3 in hSC, they were unable to detect the mRNA or protein expression of FGFR3 in tibial nerves [42]. Because the hSC used in our study were obtained from a proximal nerve segment rather than a distal peripheral nerve such as the tibial nerve, regional differences cannot be excluded. Nevertheless, together these findings suggest that FGFR3 is unlikely to play an important role as a BoNT/A (co-) receptor in hSC.

Here, it was shown that paclitaxel altered the mRNA expression of *SV2A*, *SYT1* and *SNAP25* in primary hSC. Previous studies using human and rodent tissue have demonstrated that paclitaxel induces transcriptomic and proteomic changes involving pathways related to inflammation, mitochondrial function and metabolism; however, these studies have focused mostly on neurons [33,43,44,45], whereas studies analyzing SC have examined other pain-related contexts [37,46,47]. Recently, one study evaluated for the first time the effects of paclitaxel on human-induced pluripotent stem cells (hiPSC)-derived SC [13]. Andersen reported that 48 h exposure to 0.1 μM paclitaxel resulted in 12 significantly differentially expressed genes in bulk RNA sequencing, including genes associated with lipid and lipoprotein pathways that may be associated to the myelination process [13]. These findings would contrast with our observations in primary hSC, in which *MBP* transcript levels increased under our experimental conditions. However, the two studies are not comparable, because the cellular source and model systems are not similar. Moreover, the phenotypic state of the hiPSC-derived SC was not characterized before and after paclitaxel treatment, although Andersen acknowledged that the differentiation protocol did not generate myelinating SC. Paclitaxel-induced modulation of *SV2A*, *SYT1* and *SNAP25* in primary hSC may be relevant in light of the previous findings by Marinelli et al., who reported that BoNT/A modulated SC proliferation and inhibited acetylcholine release from SC [27]. However, because the present study did not investigate protein expression, toxin binding, BoNT/A internalization or SNAP25 cleavage in primary hSC, further studies incorporating functional BoNT/A readouts are necessary to determine the physiological relevance of these transcriptional changes under basal and challenged conditions, including paclitaxel treatment in vitro.

The following limitations of the present study should be considered. The experiments were performed using cells from a single female donor and one tissue source, which limits the generalizability and does not reflect donor-to-donor, sex-related variability or tissue-source-dependent responses. Additionally, the analysis was restricted to a limited number of transcript readouts and did not assess the protein expression, cellular localization or functional endpoints. Future work should therefore validate SV2A, SYT1 and SNAP25 at the protein level and determine whether BoNT/A can cleave SNAP25 in human cells as reported in rodents [27], particularly under injury-associated conditions of clinical relevance. It will also be important to refine the phenotypic shift characterization of hSC after paclitaxel treatment by establishing hSC cultures with a more myelinating profile under basal conditions before drug exposure. Furthermore, co-culture systems combining hSC with human sensory neurons, such as hiPSC-derived sensory neurons, may be valuable to better resolve changes in hSC myelinating phenotype in a more physiologically relevant context. Including additional markers such as *MPZ* (Myelin Protein Zero), *PMP22* (Peripheral Myelin Protein 22), *JUN* (Jun proto-oncogene, AP-1 transcription factor subunit), *ATF3* (Cyclic AMP-dependent transcription factor ATF3), *EGR2* (Early Growth Response 2), *GFAP* (Glial Fibrillary Acidic Protein), *SOX10* (Transcription factor SOX10) would extend our understanding of the phenotypic remodeling, cellular adaptation and potential selective loss of immature cells under injury-associated models. Nevertheless, our findings provide an important starting point for the study of hSC plasticity and potential BoNT/A interactions in human peripheral glia.

Overall, this study provides translationally relevant transcriptional evidence that primary hSC express genes encoding BoNT/A-related molecular components and remain phenotypically responsive under paclitaxel-induced stress, as reflected by their transcriptional profile. Our findings help bridge the gap between rodent models and human peripheral glia. Further studies examining hSC responses into additional PNP models will be necessary to determine whether hSC react differently to various treatments (e.g., high glucose concentrations). If future functional studies confirm direct BoNT/A effects on hSC, our current understanding of the MoA of BoNT/A would expand beyond neurons and further support a multimodal mode of action by the toxin in the PNS.

## 4. Conclusions

This study demonstrated that untreated primary hSC express moderate levels of *NGFR* and *S100β* and low levels of *MBP* in vitro, suggesting a non-myelinating phenotype. Paclitaxel treatment induced transcriptional changes, with *NGFR* downregulated and *MBP* upregulated, suggesting phenotype switching. These responses differ from primary rat SC, possibly due to distinct initial differentiation states. The expression of *SV2A*, *SYT1*, and *SNAP25* confirmed that hSC possess transcripts encoding BoNT/A-relevant components, expanding the understanding of the MoA of BoNT/A in non-neuronal human cells. Protein-level validation and co-culture studies with sensory neurons are suggested to further assess hSC plasticity in response to injury-associated models and to better understand the role of non-neuronal cells as targets for BoNT/A.

## 5. Materials and Methods

### 5.1. Drugs, Chemicals and Materials

Paclitaxel (MedChemExpress, Cat. No. HY-B0015, Monmouth Junction, NJ, USA) was dissolved in DMSO (Sigma Aldrich, Cat. No. D2650, St. Louis, MO, USA) at 10 mM concentration stocks and stored at −80 °C. Paclitaxel was diluted in culture medium to achieve the desired experimental concentration. Figures were adapted from Servier Medical Art (https://smart.servier.com; accessed on 30 March 2026), licensed under CC BY 4.0 (https://creativecommons.org/licenses/by/4.0/; accessed on 30 March 2026).

### 5.2. Cells

Primary hSC (Female donor, Human Spinal Nerves, Lot 325I251, Innoprot, Bizkaia, Spain) were cultured and expanded, and working stocks were prepared at early stages, according to the provider’s standardized procedures under sterile/aseptic conditions (Neurosicences Innoprofile^TM^, Human Schwann Cells, Innoprot, Biskaia, Spain). Cells were cultured under well-controlled conditions on fibronectin-coated (2 μg/cm^2^) cell culture plates in Schwann Cell Medium (Cat. No. P60123, Innoprot, Biskaia, Spain) at 37 °C, 5% CO_2_. Subcultures were carried out when the culture reached 90% of confluency or above.

### 5.3. Viability of Human Schwann Cells

Primary hSC were cultured on fibronectin-coated 96-well plates at a density of 20,000 cells/cm^2^ (Passage 5, 6400 cells/well). Then, 24 h post-seeding, cells were treated with paclitaxel or vehicle (DMSO) at the indicated concentrations for 48 h. Media and treatment were refreshed after 24 h. Cell viability was evaluated using CellTiter-Glo^®^ Luminescent Cell Viability Assay, according to the manufacturer’s instructions (Promega, Cat. No. G7570, Madison, WI, USA). Bioluminescence was measured with The Infinite^®^ M1000 (Tecan, Männedorf, Switzerland), and ATP levels were quantified as an indicator of viable cells and normalized to control samples to determine the relative viability.

### 5.4. Reverse Transcription Quantitative Polymerase Chain Reaction (RT-qPCR)

hSC (Passage 4) were cultured on fibronectin-coated 12-well plates at a density of 20,000 cells/cm^2^ (68,000 cells/well). Then, 24 h post cell seedings, paclitaxel or DMSO vehicle was added, and cells were incubated with the treatment for 48 h. The treatments were refreshed after 24 h. Cells were then lysed, and RNA isolation and DNA depletion were carried out using NucleioSpin^®^ RNA Plus (Macherey-Nagel, Cat. No. 740984.250, Düren, Germany), according to the manufacturer’s instructions. RNA was quantified using The Infinite^®^ M1000 (Tecan, Switzerland). Then, 1.0 μg total RNA was reverse transcribed into cDNA with the Maxima First Strand cDNA Synthesis Kit for RT-qPCR (ThermoFisher, Cat. No. K1642, Waltham, MA, USA) in a Matercycler^®^ Nexus (Eppendorf, Hamburg, Germany) at 25 °C for 10 min followed by a 30 min at 50 °C and a 5 min at 85 °C incubation, continued by a cool-down step to 4 °C. Gene expression was determined using TaqMan^TM^ Fast Universal PCR Master Mix (2X), no AmpErase™ UNG (ThermoFisher, Cat. No. 4352042, Waltham, MA, USA) in a QuantStudio^TM^ 7 Flex Real-Time PRC System (ThermoFisher, Waltham, MA, USA); 10 μL volume/sample. All cDNA samples were diluted 1:5 in RNase-free water. Taqman FAM-MGB probes for *SV2A* (Hs01059458_m1), *SYT1* (Hs00194572_m1), *FGFR3* (Hs00179829_m1), *MBP* (Hs00921945_m1), *NGFR* (Hs00609976_m1), *S100β* (Hs00902901_m1), *SNAP25* (Hs00938957_m1) and *GAPDH* (Hs99999905_m1) were obtained from ThermoFisher Scientific (Waltham, MA, USA). PCR was conducted using an initial denaturation at 95 °C, followed by 40 amplification cycles (95 °C for 1 s, 60 °C for 20 s). For descriptive purposes only, transcript abundance was categorized based on the observed C_t_ values. Transcripts with C_t_ values < 25 were considered highly abundant, with C_t_ 25–30 moderate expression and C_t_ > 30 low abundant. The relative gene expression was normalized to *GAPDH* and determined by the 2^−ΔΔCt^ method using vehicle-treated samples as reference. Throughout this manuscript, the term p75NTR refers to the p75 neurotrophin receptor, whereas *Ngfr*/*NGFR* is used when referring to the corresponding gene.

### 5.5. Statistical Analysis

All data were plotted as the mean ± standard error of the mean (SEM). Data analysis was performed using GraphPad Prism (Version 11.0.2, San Diego, CA, USA); data sets were tested for normality using the D’Agostino–Pearson omnibus normality test. Group differences were evaluated by one-way or two-way analysis of variance (ANOVA) using the DMSO vehicle-treated group as reference, with post hoc Dunn’s or Dunnett’s multiple-comparisons tests applied where appropriate.

## Figures and Tables

**Table 1:** C_t_ values for analyzed transcripts in untreated primary hSC in vitro cultured for 24 and 48 h. Values indicate mean ± standard error of the mean. *N* ≤ 3 with technical replicates each. ntc, no template control.

Gene	C_t_ 24 h	C_t_ 48 h
*NGFR*	26.16 ± 0.09	26.33 ± 0.04
*MBP*	32.98 ± 0.08	32.58 ± 0.06
*S100β*	27.33 ± 0.04	27.62 ± 0.05
*SV2A*	27.33 ± 0.09	26.89 ± 0.05
*SYT1*	27.53 ± 0.07	27.50 ± 0.04
*SNAP25*	27.79 ± 0.04	27.68 ± 0.04
*FGFR3*	32.46 ± 0.10	32.19 ± 0.07
*GAPDH*	18.38 ± 0.06	18.19 ± 0.06
ntc	undetermined	undetermined

## Data Availability

The original contributions presented in this study are included in the article. Further inquiries can be directed to the corresponding authors.

## Supplementary Materials

The following supporting information can be downloaded at: https://www.mdpi.com/article/10.3390/toxins18080321/s1, Supplementary Table S1: Threshold cycle (Ct) values for analyzed transcripts in primary hSC after 48 h paclitaxel treatment.

## Abbreviations

The following abbreviations are used in this manuscript:

ANOVA	Analysis of Variance
BoNT/A	Botulinum Neurotoxin Type A
cDNA	Complementary Deoxyribonucleic acid
CIPN	Chemotherapy-Induced Peripheral Neuropathy
Ct	Threshold Cycle
DMSO	Dimethylsulfoxide
DRG	Dorsal Root Ganglia
FGFR3	Fibroblast Growth Factor Receptor 3
GAPDH	Glycerinaldehyde-3-Phosphate Dehydrogenase
hSC	Human Schwann Cells
iPSC	Induced Pluripotent Stem Cells
MBP	Myelin Basic Protein
p75NTR	P75 neurotrophin receptor
MoA	Mechanism of Action
NGFR	Nerve Growth Factor Receptor
Ntc	No Template Control
PCR	Polymerase Chain Reaction
PNP	Peripheral Neuropathic Pain
PNS	Peripheral Nervous System
RNA	Ribonucleic Acid
RT-qPCR	Reverse Transcription quantitative Polymerase Chain Reaction
S100β	S100 Calcium Binding Protein β
SC	Schwann Cells
SNAP25	Synaptosome Associated Protein 25
SV2A	Synaptic Vesicle Glycoprotein 2A
SYT1	Synaptotagmin 1
