# Supplementary material for: Transcriptional Profiling of Botulinum Neurotoxin Type A-Related Molecular Components in Primary Human Schwann Cells
Source: Toxins (Basel). 2026 Jul 24;18(8):321. doi: 10.3390/toxins18080321 (PMC13517366; doi:10.3390/toxins18080321)
Supplement: Supplementary file 1 [file toxins-18-00321-s001.zip › toxins-4427064-supplementary.pdf]

Supplementary Materials: Transcriptional Profiling of Botulinum Neurotoxin Type A-Related Molecular Components in Primary Human Schwann Cells

Oscar Sánchez-Carranza, Claudia Jatzke, Andreas Gravius and Jens Nagel

Supplementary Table S1. Threshold cycle (Ct) values for analyzed transcripts in primary hSC after 48 h paclitaxel treatment. Data obtained from three independent experiments (*n*=3), each performed in technical triplicate. Values are presented as mean ± SEM. Statistical comparisons were performed against the DMSO-treated condition. One-way ANOVA, Dunn's or Dunnet's test. *p=0.01, **p<0.01, ***p=0.0002, ****p < 0.0001. No-template controls (ntc) were undetermined.

Gene	CTRL	DMSO	0.001 µM	0.1 µM	10 µM
NGFR	26.33 ± 0.04	26.26 ± 0.05	26.32 ± 0.12	26.93 ± 0.04****	27.14 ± 0.08****
MBP	32.58 ± 0.06	32.66 ± 0.07	32.29 ± 0.08**	32.05 ± 0.08****	32.09 ± 0.07****
S100β	27.62 ± 0.05	27.64 ± 0.04	27.73 ± 0.06	27.60 ± 0.03	28.37 ± 0.04****
SV2A	26.89 ± 0.05	26.78 ± 0.05	26.66 ± 0.05	27.68 ± 0.05****	27.46 ± 0.03****
SYT1	27.50 ± 0.04	27.64 ± 0.03	27.52 ± 0.06	27.96 ± 0.02****	28.48 ± 0.04****
SNAP25	27.68 ± 0.04	27.54 ± 0.03	26.99 ± 0.03***	27.16 ± 0.03	27.05 ± 0.03**
FGFR3	32.19 ± 0.07	32.13 ± 0.08	31.86 ± 0.11	32.14 ± 0.04	32.61 ± 0.08*
GAPDH	18.19 ± 0.06	18.20 ± 0.04	18.23 ± 0.05	18.36 ± 0.06	18.46 ± 0.03**
ntc	undetermined	undetermined	undetermined	undetermined	undetermined
